# Analysis of the correlation between peripheral blood inflammatory markers and imaging burden of cerebral small vessel disease

**DOI:** 10.3389/fneur.2025.1538028

**Published:** 2025-07-14

**Authors:** Jiayi Yang, Zhifeng Liang, Daojing Li, Jingru Wang, Liying Mei, Liangchen Hu, Rongrong Han, Aimei Zhang, Jinfeng Ma, Liangliang Zhang, Chunxia Wang, Hongfang Li

**Affiliations:** ^1^Department of Clinical Medicine, Jining Medical University, Jining, China; ^2^Department of Neurology, Affiliated Hospital of Jining Medical University, Jining, China; ^3^Department of Hand and Foot Surgery, Affiliated Hospital of Jining Medical University, Jining, China; ^4^Health Management Center of Jining Medical University Affiliated Hospital, Jining, China

**Keywords:** cerebral small vessel disease, imaging, inflammatory factors, neutrophil count, neutrophil-lymphocyte ratio, systemic immune-inflammation index

## Abstract

**Background:**

The purpose of this study is to explore the relationship between peripheral blood inflammation-related indicators, such as neutrophil count (NC), neutrophil-to-lymphocyte ratio (NLR), and systemic immune-inflammation index (SII), and the imaging burden and subtypes of cerebral small vessel disease (CSVD).

**Methods:**

Gather relevant indicators from the population who underwent physical examinations at the Affiliated Hospital of Jining Medical University between 2018 and 2023. Evaluate the total burden and various imaging markers according to the comprehensive burden score of cerebral small vessel disease (CSVD). Employ logistic regression analysis to investigate the correlation between neutrophil count (NC), neutrophil-to-lymphocyte ratio (NLR), and systemic immune-inflammation index (SII) with the overall burden of CSVD and its distinct markers.

**Results:**

A cohort of 12,478 patients who underwent physical examinations was initially assembled. Following the application of specific inclusion and exclusion criteria, the study included 1,526 patients diagnosed with cerebral small vessel disease (CSVD) and 6,955 6,955 individuals serving as controls. Logistic regression analysis indicated no significant association between the overall burden scores of CSVD and systemic immune-inflammation index (SII), neutrophil-to-lymphocyte ratio (NIR), or neutrophil count (NC). Nonetheless, within the spectrum of imaging markers for CSVD, a significant correlation was identified between deep microbleeds and SII (*p* = 0.03), while enlarged perivascular spaces (EPVS) demonstrated a correlation with NC (*p* = 0.029, *p* = 0.019).

**Conclusion:**

This analysis reveals a correlation between NC and SII with CSCD (especially Deep cerebral microbleeds and EPVS), which has warning implications for the prevention of CSVD.

## Introduction

Cerebral small vessel disease (CSVD) is characterized by a syndrome resulting from a range of pathological changes in the brain’s small arteries, penetrating arteries, capillaries, and small veins. These alterations damage the brain’s gray and deep white matter, culminating in distinct clinical, imaging, and pathological manifestations (1). W As the population continues to age, the incidence of CSVD is rising annually, contributing to approximately 25% of ischemic stroke cases (2). About 45% of dementia is also caused by CSVD (3). The predominant clinical manifestations of CSVD encompass cognitive, motor, and emotional disturbances, as well as bowel and bladder dysfunctions (4). In recent years, there has been an annual increase in the incidence of cerebral small vessel disease (CSVD), which poses significant challenges to society and families. According to international neuroimaging standards for CSVD, the condition can be primarily categorized into five types based on imaging characteristics (5): lucanes, white matter hyperintensities (WMH), enlarged perivascular spaces (EPVS), cerebral microbleeds (CMB), brain atrophy. Recent research has identified inflammatory responses as a substantial component of the mechanisms underlying the development of CSVD (6). Currently, it is acknowledged that the principal pathophysiological mechanisms through which inflammatory markers facilitate the progression of cerebral small vessel disease (CSVD) encompass the following (1, 6, 7):(1) causing endothelial dysfunction, (2) impairing tight junctions, resulting in blood–brain barrier permeability, (3) damaging the neurovascular unit, and (4) inducing low perfusion injury. Furthermore, empirical evidence suggests that neutrophils present within the circulatory system can infiltrate and accumulate in the perivascular space, compromising the integrity of the blood–brain barrier and expediting the advancement of vascular pathologies.

Furthermore, the neutrophil-to-lymphocyte ratio (NLR) and the systemic immune-inflammation index (SII), derived from hematological laboratory markers, serve as reliable indicators of the body’s current immune status, facilitating predictions concerning the onset of associated vascular diseases (8, 9).

In this study, we examined the association between neutrophil count (NC), NLR, SII, and the overall burden of cerebral small vessel disease (CSVD), as well as each imaging biomarker of CSVD, in individuals who underwent health evaluations at Jining Medical University Affiliated Hospital over the past 5 years. This approach aims to evaluate whether there is a correlation between inflammatory markers and cerebral small vessel disease.

## Methods

### Study participants

This study gathered data from a cohort of individuals who underwent health examinations and comprehensive cranial magnetic resonance imaging at the Jining Medical University Affiliated Hospital between 2018 and 2023, encompassing a total of 12,748 participants. Exclusion criteria were applied to individuals presenting with contraindications for magnetic resonance imaging, a history of stroke, fever, or other active inflammatory or tumor-related conditions. Additionally, individuals diagnosed with Parkinson’s disease, epilepsy, severe depression, or other mental health disorders, as well as those with significant visual–spatial deficits, hearing loss, or speech impairments, were excluded from the study. This study received approval from the Ethics Committee of Jining Medical University Affiliated Hospital (IRB Approval No.: 2023-03-C005).

### Baseline clinical assessment

The demographic characteristics, risk factors (including blood pressure, body mass index, smoking history, and alcohol consumption), and medical history (encompassing hypertension, diabetes, heart disease, and hyperlipidemia) of the participants were documented at baseline. Fasting venous blood samples were obtained, and a series of assays were conducted, comprising routine blood tests, homocysteine levels, triglycerides, total cholesterol, high-density lipoprotein (HDL), and low-density lipoprotein (LDL) measurements. A comprehensive analysis of all collected data was subsequently performed.

### Blood cell count assessment

During the acquisition of medical histories, fasting venous blood samples were obtained from the study population and subsequently analyzed in the laboratory to determine neutrophil, lymphocyte, and platelet counts. Utilizing these parameters, the Neutrophil-to-Lymphocyte Ratio (NLR) and the Systemic Immune-Inflammation Index (SII), calculated as platelet count multiplied by neutrophil count divided by lymphocyte count, were derived.

### MRI acquisition and assessment

Participants underwent cranial magnetic resonance imaging (MRI) utilizing a 3.0 Tesla MRI scanner (Ingenia 3.0 T, Philips), which generated the corresponding images. The MRI protocol encompassed T1-weighted imaging (T1WI), T2-weighted imaging (T2WI), diffusion-weighted imaging (DWI), fluid-attenuated inversion recovery (FLAIR) sequences, susceptibility-weighted imaging (SWI), and magnetic resonance angiography (MRA) for cranial arterial visualization. The imaging data were systematically reviewed and analyzed by a designated reading team, consisting of two members, using the Imaging Review System. Imaging assessment was conducted following international standards for cerebral small vessel disease (CSVD) neuroimaging (5). Hyperintense signals, characterized by increased brightness on T2-weighted sequences, were identified as white matter hyperintensities (WMH). Both periventricular and deep WMH were evaluated using the Fazekas scoring criteria (10). Lacunes were observed as hypointense signals on T1-weighted sequences but appeared as hyperintense on T2 and FLAIR sequences, with diameters measuring less than 20 millimeters (11). Enlarged perivascular spaces (EPVS) were recognized as punctate or linear hyperintense signals on T2-weighted sequences, typically with diameters less than 3 millimeters, and were scored using a semi-quantitative scoring scale developed by the Edinburgh group (12). Cerebral microbleeds (CMBs) were characterized as hypointense lesions with diameters ranging from 2 to 5 millimeters on T2-weighted or susceptibility-weighted imaging (SWI) sequences and were evaluated using the CMB assessment scale (13). Given the ambiguity in evaluating brain atrophy, this study excluded brain atrophy from the imaging burden analysis. Imaging data from all included groups were randomly divided into two groups and scored and recorded by two trained evaluators (R Hang, C Hu). The evaluators maintained confidentiality regarding the participants’ clinical data. In cases of inconsistent imaging scores, evaluations were conducted by a senior neurologist who was blinded to the initial results (M Zhang). Ultimately, two evaluators assessed the overall burden of cerebral small vessel disease (CSVD) in the cranial MRI population utilizing the CSVD scoring scale developed by the Rothwell group (14). The scoring ranged from 0 to 6: 1 point was given for the presence of lacunes, CMB burden (N 1–4), EPVS (N ≥ 10), and WMH burden (Fazekas score 3–4); CMB burden (N ≥ 5) and WMH burden (Fazekas scores 5–6) were rated as 2 points.

### Statistical analysis

Categorical variables were expressed as frequencies and percentages, whereas continuous variables were presented as means and standard deviations. The baseline characteristics of participants without cerebral small vessel disease (CSVD), defined as the control population with a CSVD total burden score of 0, were compared to those with CSVD, characterized by a CSVD total burden score of 1 or greater, using the CSVD total burden scoring system developed by Rothwell et al. Categorical variables, including sex and medical history, were analyzed using the Chi-square test. Categorical variables (such as sex and medical history) were analyzed using the Chi-square test, while continuous variables (such as lymphocyte count, weight, and blood pressure) were analyzed using the Kruskal-Wallis test.

To clarify whether baseline population data (such as age, sex, blood pressure, diabetes, hypertension, laboratory test results, etc.) are correlated with the total burden of cerebral small vessel disease (CSVD) and various imaging markers, univariate analysis of variance was initially conducted between the baseline population data and the imaging markers. Considering biases due to confounding factors such as sex, age, history of hypertension, diabetes, heart disease, and differences in personal lifestyle habits (such as smoking and drinking history), we further calibrated our analysis by dividing the study population into two models. Model 1 adjusted for age and sex, while Model 2 adjusted for age, sex, blood pressure, BMI, history of hypertension, heart disease, and diabetes. We employed multivariate analysis of variance to investigate the correlation between inflammatory markers and the total burden of CSVD as well as various imaging loads in both models.

After adjusting for all confounding factors, the evaluation of NC, NLR, and SII was performed about the overall burden of cerebral small vessel disease (CSVD) and the presence and severity of various imaging markers. A multivariable logistic regression model was employed to analyze the total CSVD burden, white matter hyperintensity (WMH) burden, and cerebral microbleed (CMB) burden, with common odds ratios (cORs) and their corresponding 95% confidence intervals (CIs) being calculated. Additionally, a general logistic regression model was utilized to assess CSVD and other imaging markers of CSVD, calculating common odds ratios (ORs) along with their 95% confidence intervals (CIs).

Furthermore, the net reclassification index (NRI) and the absolute integrated discrimination improvement index (IDI) were computed to assess the impact of incorporating NC, NLR, and SII into the baseline model. The covariates included in the baseline model comprise traditional vascular risk factors associated with cerebral small vessel disease, such as age, sex, body mass index (BMI), systolic blood pressure (SBP), diastolic blood pressure (DBP), history of heart disease, diabetes, hyperlipidemia, hypertension, smoking, alcohol consumption, and levels of high-density lipoprotein (HDL), low-density lipoprotein (LDL), total cholesterol, and homocysteine. The Net Reclassification Improvement (NRI) metric is employed to measure the extent of accurate reclassification achieved through the incorporation of additional variables into the model. In contrast, the Integrated Discrimination Improvement (IDI) metric assesses the enhancement in the differentiation between events and non-events. A value of NRI or IDI greater than zero indicates an enhancement in the performance of the updated model compared to the original model (15).

The significance level for all statistical analyses in this study was set at *p* < 0.05.

All analyses in this study were performed using Empower RCH 4.1 and SPSS version 25.

## Results

### Baseline characteristics

**Figure 1:**
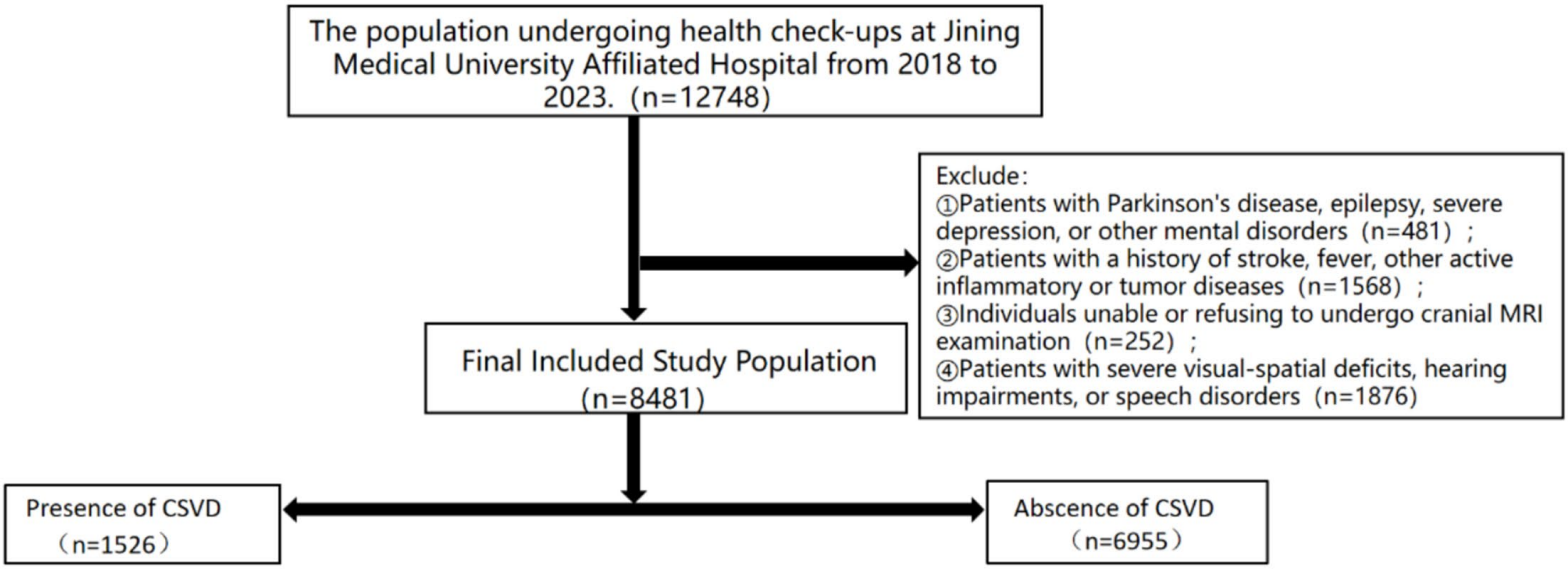
Inclusion and exclusion flowchart.

This study gathered pertinent data from a cohort of individuals undergoing medical examinations at the Jining Medical University Affiliated Hospital between 2018 and 2023, comprising a total of 12,748 participants. Exclusions were made for 481 individuals diagnosed with Parkinson’s disease, epilepsy, severe depression, or other psychiatric disorders. Additionally, 1,658 individuals were excluded due to the presence of fever, active inflammatory conditions, or neoplastic diseases. Furthermore, 252 individuals were excluded on account of significant visual–spatial deficits, auditory impairments, or speech disorders. Furthermore, a total of 1,876 participants who either declined or were unable to complete MRI scans were excluded from the study, resulting in a final sample size of 8,481 subjects. Based on the MRI imaging data, the participants were classified into Abscence of CSVD group (N = 6,955, 82%) and Presence of CSVD group (N = 1,526, 18%) as illustrated in Figure 1. Compared to the control group, the CSVD group exhibited a higher mean age, a greater proportion of female participants, and a higher prevalence of individuals with a history of hyperlipidemia, smoking, and alcohol consumption. Participants with elevated levels of hemoglobin, white blood cell count, neutrophil count (NC), neutrophil-to-lymphocyte ratio (NLR), systemic immune-inflammation index (SII), homocysteine, low-density lipoprotein, and high-density lipoprotein exhibited higher odds of cerebral small vessel disease (CSVD) (Table 1).

**Table 1:** Demographic and clinical characteristics were established according to the total burden of CSVD.

Variables	Abscence of CSVD (*n* = 6,955)	Presence of CSVD (*n* = 1,526)	*p*-value
Demographic data			
AGE, mean ± SD	54 ± 10.8	61.3 ± 10.05	<0.001
Male, *n* (%)	4,031 (57.95)	862 (56.49)	0.295
Risk factors, *n* (%)			
Hypertension	74 (1.06)	25 (1.64)	0.058
Diabetes	98 (1.41)	26 (1.70)	0.385
History of heart disease	293 (4.21)	103 (6.75)	0.052
Hyperlipidemia	515 (7.4)	148 (9.7)	<0.001
Current smoking	287 (4.13)	148 (9.7)	<0.001
Current drinking	141 (2.03)	63 (4.13)	<0.001
BMI, median (IQR) (kg/m^2^)	25.3 (14.2–35.9)	23.9 (10.8–36.7)	<0.001
SBP, mean ± SD	132 ± 19	139 ± 21	<0.001
DBP, mean±SD	81 ± 12	82 ± 13	<0.001
Laboratory data, median (IQR)			
Hemoglobin, g/L	146 (45–220)	145 (77–191)	0.027
White blood cell count (× 109/L)	5.79 (1.85–17.66)	5.83 (2.68–19.17)	0.049
Neutrophil count (× 109/L)	3.33 (0.57–13.61)	3.35 (1.09–14.08)	0.031
Lymphocyte count (× 109/L)	1.88 (0.28–10.54)	1.85 (0.64–5.64)	0.855
Platelet count (× 109/L)	243 (7–726)	240 (52–655)	0.143
NLR	1.77 (0.32–21.82)	1.79 (0.05–9.46)	0.005
PLR	128.8 (3.2–419.5)	127.6 (18.0–433.6)	0.681
SII (× 109/L)	423.6 (2.6–3565.6)	424.3 (55.6–2827.3)	0.032
HCY (mmol/L)	12.5 (3.4–93.9)	12.5 (5.2–45.6)	0.004
LDL (mmol/L)	2.95 (0.06–7.77)	2.88 (0.65–8.04)	<0.001
HDL (mmol/L)	1.33 (0.51–3.07)	1.30 (0.45–5.39)	0.022
Triglycerides (mmol/L)	1.33 (0.27–33.63)	1.33 (0.32–25.06)	0.940
Total cholesterol (mmol/L)	4.84 (1.58–15.06)	4.82 (1.98–11.88)	0.765

CSVD, cerebral small vessel disease; BMI, body mass index; IQR, interquartile range; SBP, Systolic Blood Pressure; DBP, Diastolic Blood Pressure; NLR, neutrophil-to-lymphocyte ratio; PLR, platelet-to-lymphocyte ratio; SII, systemic immune-inflammation index (platelet count × neutrophil count/lymphocyte count); HDL, high-density lipoprotein; LDL low-density lipoprotein; HCY, homocysteine.

To further elucidate the correlations, this study examines the relationship between baseline demographic data and imaging markers in the CSVD population. The analysis revealed that Age, Sex, BMI, Hyperlipidemia, and systolic blood pressure (SBP) are associated with WMH. Age, BMI, history of alcohol consumption, and HDL showed a correlation with Lacunes. Age, Sex, diabetes, and LDL exhibited a correlation with CMB. Age and BMI showed an association with EPVS. Age, gender, and systolic blood pressure (SBP) were associated with the overall burden of CSVD (*p* < 0.05) (Table 2).

**Table 2:** Univariate analysis of variance between baseline population data and total burden of CSVD as well as various imaging markers.

	WMH		Lucanes		CMB		EPVS		Total	
	OR (95% CI)	*p*	OR (95% CI)	*p*	OR (95% CI)	*p*	OR (95% CI)	*p*	OR (95% CI)	*p*
Age	1.07 (1.06, 1.08)	<0.0001	1.10 (1.06, 1.13)	<0.0001	1.0 (1.0, 1.1)	0.031	0.9 (0.9, 1.0)	<0.001	1.0 (1.0, 1.0)	<0.001
Sex, male	1.43 (1.15, 1.78)	0.0014	0.59 (0.30, 1.18)	0.1371	2.2 (1.2, 4.3)	0.016	1.3 (0.9, 1.9)	0.198	1.1 (1.0, 1.2)	0.050
BMI	0.98 (0.97, 1.00)	0.0068	0.95 (0.92, 0.97)	<0.0001	1.0 (1.0, 1.0)	0.387	1.0 (1.0, 1.1)	<0.001	1.0 (1.0, 1.0)	0.386
Hypertension	1.25 (0.91, 1.73)	0.1694	0.91 (0.32, 2.60)	0.8614	1.1 (0.4, 2.8)	0.828	1.0 (0.5, 2.0)	0.886	1.0 (0.9, 1.2)	0.579
Diabetes	1.32 (0.83, 2.09)	0.2429	0.35 (0.12, 1.02)	0.0547	3.4 (1.4, 8.0)	0.005	1.4 (0.6, 3.3)	0.456	1.1 (0.9, 1.3)	0.451
History of heart disease	0.86 (0.36, 2.08)	0.7381	0.61 (0.08, 4.60)	0.6288	1.3 (0.2, 9.5)	0.792	1.8 (0.5, 6.1)	0.348	1.0 (0.7, 1.4)	0.855
Hyperlipidemia	2.02 (1.14, 3.60)	0.0163	0.015 (0.01, 0.02)	0.411	1.3 (0.2, 9.1)	0.823	1.1 (0.3, 4.7)	0.913	1.2 (0.9, 1.6)	0.254
Current smoking	1.33 (0.92, 1.92)	0.1335	0.84 (0.25, 2.78)	0.7760	2.0 (0.9, 4.8)	0.107	1.8 (1.0, 3.4)	0.069	1.1 (0.9, 1.3)	0.382
Current drinking	1.02 (0.72, 1.43)	0.9193	0.42 (0.19, 0.93)	0.027	1.0 (0.4, 2.6)	0.937	1.1 (0.6, 2.0)	0.797	1.0 (0.9, 1.1)	0.912
SBP	1.02 (1.01, 1.02)	<0.001	1.01 (1.00, 1.03)	0.0767	1.0 (1.0, 1.0)	0.100	1.0 (1.0, 1.0)	0.096	1.0 (1.0, 1.0)	0.005
DBP	1.00 (1.00, 1.01)	0.4128	1.00 (0.98, 1.03)	0.9949	1.0 (1.0, 1.0)	0.416	1.0 (1.0, 1.0)	0.801	1.0 (1.0, 1.0)	0.654
HCY	1.00 (0.98, 1.02)	0.7360	0.95 (0.91, 1.00)	0.0719	1.0 (1.0, 1.1)	0.509	1.0 (1.0, 1.1)	0.075	1.0 (1.0, 1.0)	0.755
NC	1.00 (0.91, 1.08)	0.9123	0.90 (0.71, 1.13)	0.3537	1.1 (0.8, 1.3)	0.628	0.9 (0.8, 1.1)	0.261	1.0 (1.0, 1.0)	0.774
NLR	1.09 (0.98, 1.21)	0.1056	1.08 (0.74, 1.58)	0.6911	1.2 (0.9, 1.5)	0.279	0.9 (0.7, 1.2)	0.453	1.0 (1.0, 1.1)	0.478
SII	1.00 (1.00, 1.00)	0.5497	1.00 (1.00, 1.00)	0.4516	1.0 (1.0, 1.0)	0.518	1.0 (1.0, 1.0)	0.970	1.0 (1.0, 1.0)	0.648
LDL	0.92 (0.81, 1.05)	0.1985	1.00 (0.67, 1.49)	0.9953	0.6 (0.4, 0.9)	0.006	1.2 (0.9, 1.5)	0.158	1.0 (0.9, 1.0)	0.443
HDL	1.06 (0.80, 1.41)	0.6840	3.94 (1.18, 13.12)	0.0255	0.9 (0.4, 2.0)	0.759	0.8 (0.5, 1.5)	0.553	1.0 (0.9, 1.1)	0.932
Total cholesterol	0.95 (0.86, 1.05)	0.3350	0.96 (0.70, 1.31)	0.7777	0.7 (0.5, 0.9)	0.017	1.1 (0.9, 1.3)	0.226	1.0 (0.9, 1.0)	0.589
Triglyceride	0.97 (0.90, 1.05)	0.4622	0.92 (0.81, 1.04)	0.1984	1.0 (0.8, 1.2)	0.980	1.0 (0.9, 1.1)	0.578	1.0 (1.0, 1.0)	0.751

This table presents the 95% confidence intervals of common odds ratios (cORs) between baseline population collected data (such as sex, age, blood pressure, BMI, etc.) and the total burden of CSVD as well as various imaging burdens. Total: total burden of CSVD; BMI: body mass index; SBP: Systolic Blood Pressure; DBP: Diastolic Blood Pressure; NC: neutrophil count; NLR: neutrophil-to-lymphocyte ratio; SII: systemic immune inflammation index (calculated as platelet count × neutrophil count/lymphocyte count); HDL: high-density lipoprotein; LDL: low-density lipoprotein; HCY: homocysteine.

To adjust for confounding factors such as age, sex, blood pressure, BMI, and medical history, we divided the experimental group into two models: Model 1 adjusted for age and sex; Model 2 adjusted for age, sex, BMI, Hypertension, Diabetes, Heart disease, Hyperlipidemia, smoking history, and alcohol history, and conducted multifactorial analysis of variance with respect to the total burden of CSVD and each imaging marker. The results indicated no statistically significant differences (*p* > 0.05) (Table 3).

**Table 3:** Multivariate analysis of variance for inflammatory markers and imaging markers.

	SII				NC				NLR			
	Model 1		Model 2		Model 1		Model 2		Model 1		Model 2	
	OR (95% CI)	*p*	OR (95% CI)	*p*	OR (95% CI)	*p*	OR (95% CI)	*p*	OR (95% CI)	*p*	OR (95%CI)	*p*
WMH	1.00 (1.00, 1.00)	0.79	1.00 (1.00, 1.00)	0.99	0.97 (0.89, 1.06)	0.49	0.96 (0.88, 1.05)	0.35	1.01 (0.91, 1.13)	0.81	1.00 (0.89, 1.11)	0.97
CMB	1.00 (1.00, 1.00)	0.61	1.00 (1.00, 1.00)	0.48	1.01 (0.81, 1.27)	0.90	0.99 (0.78, 1.25)	0.93	1.08 (0.82, 1.42)	0.60	1.08 (0.80, 1.45)	0.62
EPVS	1.00 (1.00, 1.00)	0.90	1.00 (1.00, 1.00)	0.86	0.89 (0.75, 1.06)	0.20	0.87 (0.72, 1.04)	0.13	0.92 (0.73, 1.17)	0.50	0.92 (0.72, 1.18)	0.52
Lucanes	0.00 (−0.00, 0.00)	0.39	0.00 (−0.00, 0.00)	0.38	−0.00 (−0.01, 0.00)	0.45	−0.00 (−0.01, 0.00)	0.55	0.00 (−0.01, 0.01)	0.75	0.00 (−0.01, 0.01)	0.74
Total	1.00 (1.00, 1.00)	0.68	1.00 (1.00, 1.00)	0.73	0.99 (0.95, 1.03)	0.54	0.99 (0.95, 1.02)	0.49	1.00 (0.96, 1.05)	0.88	1.00 (0.95, 1.05)	0.95

This table demonstrates the 95% confidence intervals for the common odds ratios (cORs) of inflammatory markers, total burden of CSVD, and various imaging burdens after adjusting for confounding factors. Model 1: adjusted for age and sex; Model 2: adjusted for age, sex, BMI, medical history (including Heart disease, Hiabetes, Hypertension, Hyperlipidemia), smoking history, alcohol consumption history, Homocysteine, Blood pressure, Triglycerides, et al. Total: total burden of CSVD; NC: neutrophil count; NLR: neutrophil-to-lymphocyte ratio; SII: systemic immune inflammation index (calculated as platelet count × neutrophil count/lymphocyte count).

### Association of NC, NLR, and SII with CSVD

**Figure 2:**
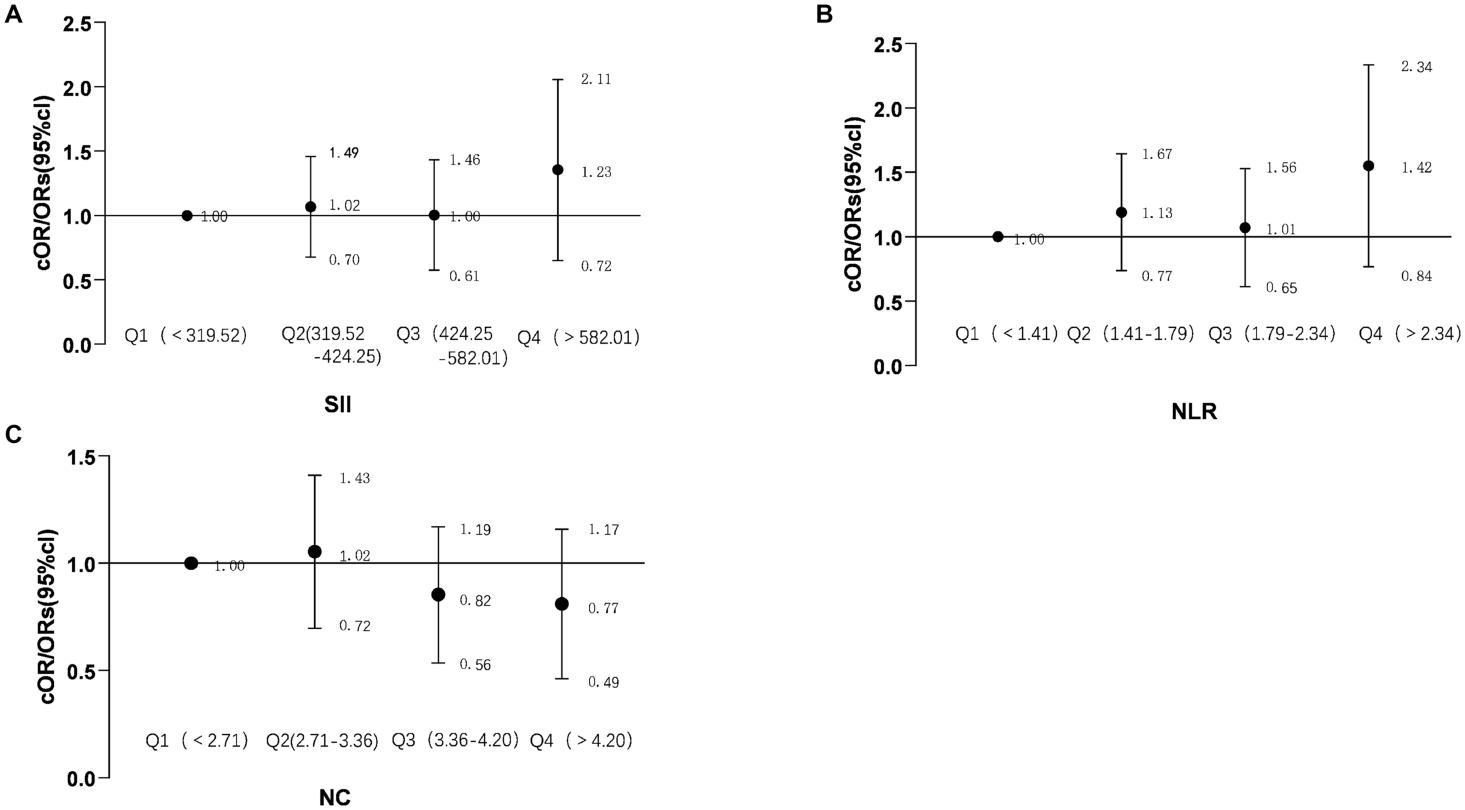
Forest plot showing the correlation between NC, NLR, SII, and the total burden of CSVD. The forest plot illustrates the common odds ratios (cORs) for NC about the total burden of CSVD or the presence of CSVD **(A)**; SII (Systemic Immune Inflammation Index) and its cORs related to the total burden of CSVD or the presence of CSVD **(B)**; NLR (Neutrophil-to-Lymphocyte Ratio) and its cORs regarding the total burden of CSVD or presence of CSVD **(C)**. The relationship between the total burden of CSVD and ordinal categorical variables is represented by cORs. The black line indicates the 95% confidence intervals for the cORs. NC, neutrophil count; NLR, neutrophil-to-lymphocyte ratio; SII, systemic immune inflammation index (calculated as platelet count × neutrophil count/lymphocyte count); cORs, common odds ratio; Total burden of CSVD, includes the presence of lacunar infarcts, periventricular space (more than 10), 1–4 microbleeds, and moderate white matter hyperintensity (Fazekas score of 3–4 from periventricular and subcortical white matter), scoring 1 point; more than 5 microbleeds and severe white matter hyperintensity (Fazekas score of 5–6 from periventricular and subcortical white matter) score 2 points.

To investigate the association between NC, NLR, and SII and the overall burden of CSVD, the imaging data of individuals in the CSVD group were analyzed using the CSVD total burden scoring criteria developed by Rothwell. To enhance the precision of the correlation analysis, participants in the CSVD cohort were stratified into four groups according to quartiles of NC, NLR, and SII. A logistic regression model was employed for the statistical analysis. The results of this study reveal that NC, NLR, and SII do not exhibit a correlation with the overall burden of CSVD (P>0.05), as illustrated in Figure 2.

**Figure 3:**
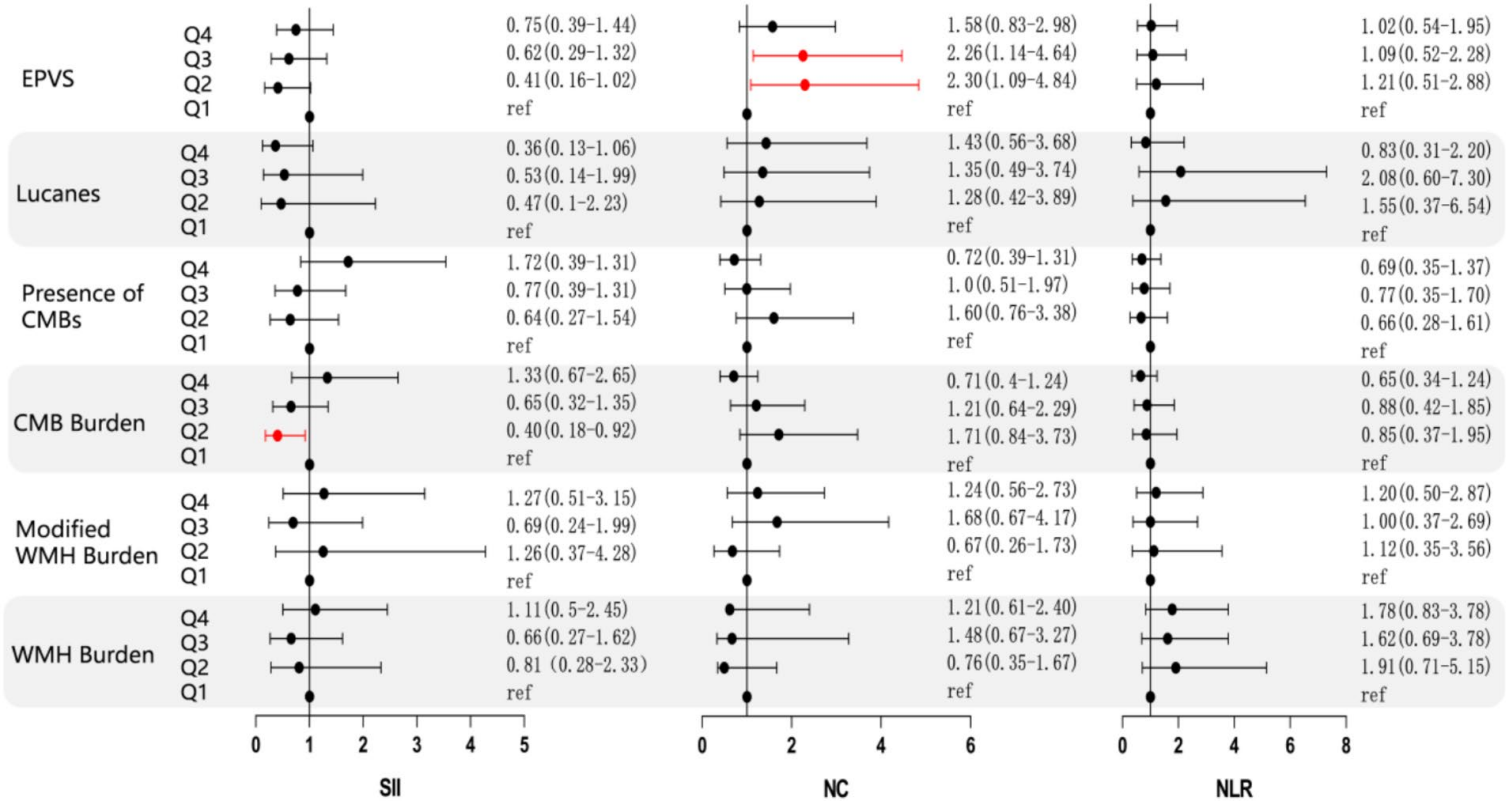
Forest plot illustrating the correlation between NC, NLR, SII, and various imaging markers of CSVD. The forest plot displays the common odds ratios (cORs) for SII, NC, and NLR about WMH, Lucanes, CMBs, and EPVS as assessed by the Rothwell scoring criteria. The correlations for WMH burden and CMB burden are represented using cORs, while results for other markers are expressed as ORs. The black line represents the 95% confidence intervals for the cORs/ORs. *NC*: neutrophil count; *NLR*: neutrophil-to-lymphocyte ratio; *SII*: systemic immune inflammation index (platelet count × neutrophil count/lymphocyte count); *cOR*: common odds ratio; *OR*: odds ratio. WMH burden is classified as grade 0: total periventricular + subcortical white matter hyperintensity rated 1–2 points; Grade 1: total periventricular + subcortical white matter hyperintensity rated 3–4 points; Grade 2: total periventricular + subcortical white matter hyperintensity rated 5–6 points. CMBs burden is categorized as grade 0: none; grade 1: 1–4 microbleeds; grade 2: ≥5 microbleeds. EPVS is defined as periventricular spaces greater than 10.

After conducting a multifactorial analysis of variance, it was found that, after adjusting for confounding factors, NC, NLR, and SII showed no significant correlation with the imaging burden of CSVD. Therefore, we categorized the inflammatory markers into quartiles and conducted a more detailed scoring stratification of the imaging burden, using binary and multinomial logistic regression models for statistical analysis of the two factors. This categorization aimed to evaluate the correlation of each inflammatory marker with various imaging burdens of CSVD, including lacunes, WMH burden, modified WMH burden, CMB burden, presence of CMBs, and EPVS. The findings indicate that, specifically in deep CMBs, the second quartile of SII demonstrated a correlation. The analysis revealed a significant correlation between the presence of deep CMBs and the second quartile of SII (OR 0.4, 95%CI 0.18–0.92, P_Q2_ = 0.03). Furthermore, in the context of EPVS, both the second and third quartiles of NC demonstrated significant correlations with their presence (OR 2.30, 95%CI 1.09–4.84, P_Q2_ = 0.029; OR 2.26, 95%CI 1.14–4.64, P_Q3_ = 0.019), as illustrated in Figure 3. However, there were no significant differences between SII and WMHs, presence of CMBs (CMB count > 5), and Lucanes; there were also no significant differences between NC and WMHs, CMBs, Lucanes, and between NLR and WMHs, CMBs, Lucanes, and EPVS (Figure 3).

### Improvement in the prediction model for CSVD by the addition of NC, NLR and SII

We conducted a comparative analysis of various models to assess their efficacy in predicting the presence of cerebral small vessel disease (CSVD), as detailed in Table 2. The findings indicate that the integration of neutrophil-to-lymphocyte ratio (NLR), neutrophil count plus NLR, NC plus systemic immune-inflammation index (SII), and SII plus NLR into the baseline model enhanced the net reclassification improvement (NRI) for predicting CSVD. The NRI values were 3.67% (95% CI: 2.73, 4.61%), 2.49% (95% CI: 1.71, 3.27%), 4.33% (95% CI: 3.30, 5.35%), and 0.26% (95% CI: −0.59, 1.11%), respectively. Notably, the model incorporating NC was the only one to exhibit divergent predictive outcomes for NRI and integrated discrimination improvement (IDI), while all other models showed enhanced performance across both metrics. In the predictive model incorporating NC, a marginal decline in NRI performance was observed, resulting in an NRI value of −0.85% (95% CI: −1.49, 0.21%). Conversely, the IDI showed a slight enhancement in predictive capability, with an IDI value of 0.05% (95% CI: −0.66, 0.77%). Furthermore, in the two predictive models that included SII and the combination of NC, NLR, and SII, there was no significant alteration in NRI values compared to previous assessments. However, the IDI demonstrated a modest improvement in predictive performance, with values of 0.45% (95% CI: −0.41, 1.3%) and 0.26% (95% CI: −0.66, 1.18%), respectively (see Table 4).

**Table 4:** The NRI and IDI estimate of NC, NLR, and SII.

Variables	NRI		IDI	
	Estimate (95% CI), %	*p* value	Estimate (95% CI), %	*p* value
Presence of CSVD				
Basic model	ref		ref	
Basic model+ NC	−0.85(−1.49,-0.21)	0.0092	0.05 (−0.66, 0.77)	0.8818
Basic model+ NLR	3.67(2.73,4.61)	< 0.0001	−0.07 (−1.11,0.97)	0.8959
Basic model+ NC + NLR	2.49 (1.71, 3.27)	< 0.0001	0.15 (−0.72, 1.01)	0.7399
Basic model+ NC + SII	4.33 (3.30, 5.35)	< 0.0001	0.56 (−0.56, 1.67)	0.3275
Basic model+ SII + NLR	0.26 (−0.59. 1.11)	0.5464	0.35 (−0.58, 1.28)	0.4635

NC neutrophil count, NLR neutrophil-to-lymphocyte ratio, SII systemic immune inflammation index (platelet count × neutrophil count/lymphocyte count), NRI net reclassification index, IDI integrated discrimination improvement. Basic model: multivariable logistic regression model included age, sex, body mass index, diabetes, hypertension, total cholesterol, high-density lipoprotein, low-density lipoprotein, homocysteine, previous dyslipidemia, previous heart disease, current smoking, current drinking.

## Discussion

This study examined the relationship between neutrophil count (NC), neutrophil-to-lymphocyte ratio (NLR), and systemic immune-inflammation index (SII) with the overall burden of cerebral small vessel disease (CSVD) and various imaging markers in individuals undergoing health check-ups at Jining Medical University Affiliated Hospital from 2018 to 2023. Statistical analysis found no significant correlation between NC, NLR, and SII with the overall burden of CSVD. However, among the imaging markers of CSVD, a positive correlation was found between EPVS and NC, as well as between CMB (especially CMB burden) and SII. In the basic models, the addition of NC and NLR, NC and SII, as well as a new model incorporating SII and NLR led to improvements in the NRI values, while the IDI value was positive, indicating that any two indicators interacting can predict the presence of CSVD. In conclusion, our study demonstrates that the increase in inflammatory markers is associated with a higher prevalence of CSVD. Interactions among neurons, blood vessels, and inflammatory cells are essential for maintaining normal brain function; disruptions in these interactions may contribute to chronic pathological inflammation (1). Neutrophils, lymphocytes, and platelets are crucial constituents of the human immune system, rapidly responding to internal and external insults by initiating a cascade of inflammatory responses (16, 17). Research has demonstrated that the neutrophil-to-lymphocyte ratio (NLR) effectively reflects subclinical inflammatory states (9, 18). However, the study found no correlation between NLR and the imaging burden of CSVD. As shown in Table 1, the NLR values in the CSVD group were generally lower than those in the healthy control group, while previous studies have indicated that the NLR values are higher in both stroke and CSVD populations. Consequently, we infer that this result may have been influenced by the lower NLR values in the enrolled study population. Previous studies have established a correlation between elevated levels of neutrophil count (NC), NLR, and SII with the incidence, severity, and unfavorable prognosis in stroke patients (19, 20). This study did not identify a significant correlation between inflammatory markers and the overall burden of cerebral small vessel disease (CSVD). Previous research demonstrated a notable association between elevated neutrophil-to-lymphocyte ratio (NLR) and systemic immune-inflammation index (SII) with changes in the white matter among COVID-19 patients (21). In contrast, the present study found that increased levels of neutrophil count (NC) and SII were positively associated with specific imaging markers of CSVD, particularly cerebral microbleeds (CMB) and enlarged perivascular spaces (EPVS). Consequently, it can be inferred that inflammatory markers may serve as potential risk indicators for subclinical CSVD, particularly for CMB and EPVS.

The precise mechanisms through which neutrophil count (NC), neutrophil-to-lymphocyte ratio (NLR), and systemic immune-inflammation index (SII) contribute to cardiovascular and cerebrovascular injury remain inadequately understood. Neutrophils, lymphocytes, and platelets play significant roles in immune regulation (22). Chronic inflammation can stimulate the adhesion of white blood cells, platelets, and other inflammatory cells to the vascular endothelium, leading to endothelial dysfunction (23). Simultaneously, inflammatory mediators, including lymphocytes and neutrophils, infiltrate the perivascular spaces, disrupting the blood–brain barrier and expanding these spaces. This process facilitates the release of inflammatory agents such as tumor necrosis factor and interleukins, thereby accelerating the inflammatory cascade, which ultimately results in white matter pathology (24, 25). Additionally, conditions such as hypertension, diabetes, and hyperlipidemia may induce a series of inflammatory responses that contribute to the onset of cerebral small vessel disease (CSVD) (6). The inflammatory responses contributing to CSVD are highly complex, and no definitive conclusions have been reached; further research is required.

Nonetheless, this study possesses certain limitations. The absence of variable adjustment analyses to control for confounding factors may introduce potential sources of error in the results. Furthermore, during the face-to-face data collection for the baseline population, the investigation into participants’ medication histories was not conducted. As a result, it is not possible to rule out the possibility that some participants may have exhibited reduced inflammatory markers in their blood due to medication use, which could compromise the accuracy of the findings. In conclusion, it is crucial to acknowledge that the majority of participants in this study were sourced from a single city in China. This sampling approach may introduce selection bias and potentially constrain the generalizability of the research findings.

## Conclusion

This study concludes that within the population undergoing evaluations, inflammatory markers in the blood, specifically the Systemic Immune-Inflammation Index (SII) and Neutrophil Count (NC), are correlated with cerebral small vessel disease (CSVD), particularly cerebral microbleeds (CMB) and enlarged perivascular spaces (EPVS). It is hypothesized that SII and NC serve as significant indicators for predicting the presence and progression of CSVD. The implementation of early preventive strategies for individuals exhibiting elevated levels of SII or NC may potentially reduce the incidence and progression of CSVD.

## Data Availability

The original contributions presented in the study are included in the article/supplementary material, further inquiries can be directed to the corresponding author.
